# Hydrophobic Acrylic Trifocal IOL in Post–Laser Refractive Surgery Eyes: Visual Performance, Predictability, and Patient‐Reported Outcomes

**DOI:** 10.1155/joph/7762608

**Published:** 2026-10-21

**Authors:** Yong Hyu Goh, Yoon Seong Choi, Sungho Choi, Dong Hui Lim, Sung Jin Na, Deok Jo Nam

**Affiliations:** ^1^ Department of Ophthalmology Samsung Medical Center Sungkyunkwan University School of Medicine 81 Irwon-ro, Gangnam-gu Seoul 06351 South Korea skku.edu; ^2^ The New York University College of Arts and Science 70 Washington Square South New York 10012 New York USA; ^3^ First Samsung Eye Clinic 22, Seocho-daero 78-gil, Seocho-gu Seoul South Korea; ^4^ Samsung Advanced Institute for Health Sciences and Technology Sungkyunkwan University Seoul South Korea skku.edu

**Keywords:** Clareon PanOptix, corneal higher-order aberrations, IOL power calculation, patient satisfaction, post-laser refractive surgery, total keratometry, trifocal intraocular lens

## Abstract

**Purpose:**

To evaluate clinical and patient‐reported outcomes of the Clareon PanOptix (CP) hydrophobic acrylic trifocal IOL in eyes with previous myopic laser refractive surgery (LRS).

**Methods:**

This single‐center, ambispective study included 94 eyes of 47 patients with previous myopic LRS undergoing CP implantation. At 6 months, monocular visual acuity (VA), refraction, and refractive outcomes were assessed. Prediction accuracy was compared using Barrett True‐K and Haigis‐L with conventional keratometry (CK) or total keratometry (TK) on the IOLMaster 700 and Barrett True‐K with CK or TK on the Argos; Argos TK incorporated Pentacam‐measured posterior corneal astigmatism. Spectacle dependence, satisfaction, and visual disturbances were assessed. Correlations between preoperative corneal higher‐order aberrations (HOAs) and postoperative outcomes were analyzed.

**Results:**

Mean uncorrected VAs were 0.028 ± 0.087 logMAR for distance, 0.177 ± 0.130 logMAR for intermediate, and 0.093 ± 0.137 logMAR for near. The mean postoperative spherical equivalent was −0.142 ± 0.375 D, with 81% of eyes within ±0.50 D of emmetropia. In a retrospective comparison of IOL power calculation methods, Barrett True‐K with TK on the IOLMaster 700 yielded the highest observed proportion of eyes within ±0.50 D of the intended target (78.7%). After Holm–Bonferroni correction, preoperative corneal HOA RMS and nonspherical HOA RMS at a 3.0‐mm diameter were associated with uncorrected distance VA, whereas corneal HOAs at larger analysis diameters (5.0–6.0 mm) were associated with postoperative visual disturbances. Patients reported high satisfaction (3.09 ± 0.62) and complete spectacle independence for distance and intermediate vision.

**Conclusions:**

The CP trifocal IOL is an effective option for presbyopia correction in post‐myopic LRS eyes meeting predefined preoperative corneal aberration criteria. Refractive predictability depended on device‐specific formula–keratometry combinations, with Barrett True‐K yielding the highest observed ±0.50 D rates with TK (IOLMaster 700) and with CK (Argos). Corneal HOA analysis at specified measurement diameters may further guide patient selection and counseling.

## 1. Introduction

An increasing number of patients who underwent laser‐assisted in situ keratomileusis (LASIK) or photorefractive keratectomy (PRK) in their youth are now developing cataracts and thus requiring cataract surgery [1]. Having experienced long‐term spectacle independence, these patients often expect to maintain high‐quality uncorrected vision postoperatively [2, 3].

Multifocal intraocular lenses (IOLs) have emerged as a viable option to meet these expectations [4]. However, the use of multifocal IOLs in eyes with a history of laser refractive surgery (LRS, including LASIK or PRK) remains controversial [5, 6]. The principal concerns include difficulties in accurate IOL power calculation in eyes with altered corneal anatomy and the increased risk of adverse visual phenomena such as reduced contrast sensitivity, glare, and halos following multifocal IOL implantation [7, 8]. It is well established that corneal spherical aberration (SA) and higher‐order aberrations (HOAs), including coma and trefoil, are significantly increased after myopic LASIK and PRK [9, 10]. Because corneal HOAs play a crucial role in affecting postoperative visual quality after multifocal IOL implantation, preoperative evaluation of HOAs is essential [9, 11]. Nevertheless, several studies have reported favorable visual outcomes and high levels of patient satisfaction in post‐LRS eyes implanted with multifocal IOLs, including the AcrySof IQ PanOptix (AP; Alcon, Fort Worth, TX, USA), suggesting that multifocal IOLs may remain a viable option in carefully selected patients who undergo a thorough preoperative assessment [1, 12].

The AP platform is based on a modified quadrifocal diffractive design that technically generates four focal points. However, the light that would form a 120‐cm focal point is redirected to the distance focus, so that the lens functions as a trifocal IOL providing near, intermediate, and distance vision [13]. The Clareon PanOptix (CP; Alcon, Fort Worth, TX, USA) shares the same optical design as the AP, with the principal difference being the IOL material. Although the AP is made of the AcrySof hydrophobic acrylic copolymer (phenylethyl acrylate/phenylethyl methacrylate), the CP uses the newer Clareon hydrophobic acrylic material incorporating hydroxyethyl methacrylate (HEMA), which is designed to reduce material‐related optical phenomena such as glistening formation and surface haze reported with AcrySof IOLs [14–17]. Despite these material refinements, to the best of our knowledge, no published studies have specifically evaluated the clinical outcomes of CP implantation in post‐LRS eyes. Moreover, most existing studies have focused primarily on visual acuity (VA) and refraction outcomes, while detailed investigations into the underlying causes of poor visual quality have been relatively limited.

The primary purpose of this study was to determine the clinical efficacy, safety, and refractive predictability of the CP trifocal IOL in eyes with previous myopic LRS. Secondary aims were to comprehensively assess patient‐reported outcomes—including spectacle independence, satisfaction, and visual disturbances—via standardized questionnaires, and to examine the relationship between these subjective outcomes and preoperative corneal HOAs by means of a multidiameter analytical approach.

## 2. Methods

### 2.1. Study Design

This ambispective, single‐arm study enrolled patients who underwent CP implantation at the First Samsung Eye Clinic (Seoul, South Korea) between November 2021 and June 2023. Clinical data were retrospectively reviewed from medical records, while patient‐reported outcomes were prospectively collected via standardized questionnaires. All surgical and diagnostic procedures were performed at a single center using uniform examination protocols and surgical techniques that remained unchanged throughout the study period. This study adhered to the tenets of the Declaration of Helsinki and was approved by the Institutional Review Board of First Samsung Eye Clinic (IRB No. FSEC‐202311‐HR010‐02). All participants provided written informed consent for the prospective component.

### 2.2. Patients

All patients had a history of myopic LRS before cataract surgery. Because reliable pre‐LRS refractive records were not consistently available, surgical eligibility and the presence of a myopic ablation pattern were determined on the basis of current corneal morphology assessed by Scheimpflug‐based corneal tomography (Pentacam HR; Oculus, Wetzlar, Germany). Attention was given to the Holladay Equivalent Keratometry Readings (EKR) Detail Report to confirm a myopic ablation profile, and to the axial, sagittal, and elevation maps to evaluate optical zone centration and symmetry.

Only eyes with an overall regular corneal topographic pattern on preoperative corneal tomography were included. Corneal HOAs were measured preoperatively using Scheimpflug‐based corneal tomography. Eyes were included if (i) the preoperative primary SA within the 4.0‐mm analysis diameter on Pentacam tomography ranged from −0.01 to 0.18 μm and (ii) coma values were within ±3 SD of the normalized Zernike values provided by the Pentacam. In addition, the total corneal HOA root mean square (RMS) was required to be < 0.6 μm within a 4.0‐mm analysis diameter. These aberration criteria are part of our institution’s routine preoperative screening protocol, applied at the time of IOL selection to all candidates for multifocal IOL implantation. The criteria were established based on our previous clinical experience, in which VA improved after normalization of corneal SA by topography‐guided LASIK in patients dissatisfied after multifocal IOL implantation [18], and on clinical observations of poorer postoperative visual quality following multifocal IOL implantation outside these ranges. Exclusion criteria were corneal opacity, tomographic evidence of optical zone decentration, irregular astigmatism, asymmetric elevation patterns, or features suggestive of early corneal ectasia on Pentacam imaging, as well as moderate‐to‐severe dry eye disease, retinal pathology, glaucoma, or a history of corneal transplantation.

All enrolled patients underwent comprehensive preoperative evaluation, including uncorrected distance visual acuity (UDVA), corrected distance visual acuity (CDVA), manifest refraction, intraocular pressure measurement, slit‐lamp microscopy, specular microscopy (CEM‐530, Nidek Co., Ltd., Gamagori, Japan), ocular biometric measurements with the IOLMaster 700 (Carl Zeiss Meditec Inc., Jena, Germany) and Argos (Alcon, Fort Worth, TX, USA), ultra‐wide‐field fundus photography (Daytona, Optos plc, Dunfermline, UK), and macular examination using spectral‐domain optical coherence tomography (Cirrus OCT 5000; Carl Zeiss Meditec Inc., Jena, Germany). The target postoperative spherical equivalent (SE) was set at emmetropia or minimal hyperopia in the dominant eye and emmetropia or minimal myopia in the nondominant eye. Toric IOLs were implanted when preoperative corneal astigmatism was ≥ 0.50 diopters (D) to minimize residual postoperative refractive error.

### 2.3. Outcome Measures

Clinical outcomes were evaluated at 1, 3, and 6 months postoperatively. Monocular VA assessments included UDVA and CDVA at 4 m; uncorrected intermediate visual acuity (UIVA) and distance‐corrected intermediate visual acuity (DCIVA) at 60 cm; and uncorrected near visual acuity (UNVA) and distance‐corrected near visual acuity (DCNVA) at 40 cm. All VA values were recorded in decimal units and converted to logMAR. Near and intermediate vision were measured with the LEA‐Numbers under photopic conditions (85 cd/m^2^). Subjective refraction was assessed by two experienced optometrists via retinoscopy and trial lenses.

Prediction error (PE) was calculated at 6 months as the postoperative SE minus the predicted refraction for the implanted IOL power, using predictions from two biometers—the IOLMaster 700 and Argos. With the IOLMaster 700, predicted refraction was obtained using the Barrett True‐K and Haigis‐L formulas with both total keratometry (TK) and conventional keratometry (CK). With the Argos, the Barrett True‐K formula was used with CK and TK. For the Argos analysis, the term “Barrett True‐K with TK” refers to the Barrett True‐K calculation in which posterior corneal astigmatism obtained from Pentacam was incorporated, as the Argos does not provide a native TK measurement. Biometric parameters such as axial length (AL), anterior chamber depth (ACD), lens thickness (LT), and keratometry (K) were also compared between the two devices. K and TK were analyzed using each biometer’s native refractive index model and device‐specific formulas, without standardization across devices. The refractive indices applied were 1.3375 for the IOLMaster 700 and, for the Argos, 1.376 for the cornea, 1.410 for the lens, and 1.336 for the aqueous humor and vitreous. ULIB‐recommended IOL constants were used for the IOLMaster 700, whereas institution‐optimized constants were applied for the Argos.

At the 6‐month follow‐up, patients completed QUVID Ver.B (visual disturbances and spectacle dependence) and IOLSAT Ver.B (overall satisfaction). Visual disturbances were graded on a 5‐point scale (0 = no discomfort to 4 = always). Spectacle dependence was evaluated using a 5‐point scale (0 = no use at all to 4 = always), and overall satisfaction with vision was rated on a 5‐point scale (0 = very dissatisfied to 4 = very satisfied). These outcomes were stratified by visual task—near (reading and smartphone use), intermediate (computer use), and distance (driving and outdoor activities)—and assessed under various lighting conditions. Spectacle dependence for near vision was assessed both monocularly and under binocular viewing conditions.

Preoperative corneal HOAs were quantified using the Pentacam at analysis diameters of 3.0 to 6.0 mm in 1.0‐mm increments (Supporting Table 1). Relationships between preoperative HOAs and postoperative VA, patient satisfaction, and visual disturbances were analyzed (Supporting Table 2). HOA RMS was used as a global index of total corneal HOAs. Primary SA (Z_4_^0^) represented the rotationally symmetric component of corneal HOAs reflecting the aspheric profile of the cornea. Nonspherical corneal HOA RMS (NS‐HOA RMS) was defined as the residual HOA magnitude after excluding SA. This metric predominantly reflects nonspherical (largely asymmetric) aberrations such as coma and trefoil.

### 2.4. Surgical Technique

Cataract surgeries were performed by two experienced surgeons under proparacaine HCl 0.5% (Alcaine, Alcon) using phacoemulsification (Legion, Alcon) through a 2.5‐mm clear corneal incision. A digital marker microscope system (DMM Image Guided System, Alcon) was used to assist corneal incision localization, continuous curvilinear capsulorhexis, and toric IOL alignment.

### 2.5. Statistical Analysis

Statistical analyses were performed using SPSS v28.0 (IBM Corp., Armonk, NY, USA). Normality was assessed with the Shapiro–Wilk test. For normally distributed data, the paired *t*‐test and Pearson’s correlation coefficient were used. For non‐normally distributed data, the Wilcoxon signed‐rank test and Spearman’s rank correlation coefficient (*ρ*) were applied. To control the family‐wise error rate and account for multiple comparisons, *p*‐values were adjusted using the Holm–Bonferroni stepdown procedure. Descriptive statistics were reported as mean ± SD or percentage, and a *p*‐value of < 0.05 was considered statistically significant.

## 3. Results

A total of 94 eyes from 47 patients were included in this study, including 14 males (29.8%). The mean age was 53.70 ± 5.28 years (range, 45–66 years). Twenty‐eight patients (56 eyes) had previously undergone LASIK, whereas the other 19 patients (38 eyes) had received PRK. Preoperative data, including VA, refraction, and ocular biometry from both devices, are presented in Table 1.

**TABLE 1:** Preoperative visual acuity, refractive error and biometric data.

	**Mean ± SD [range]**		

Sphere (D)	−0.50 ± 1.20 [−5.00, 1.50]		
Cylinder (D)	−0.42 ± 0.45 [−1.50, 0.00]		
SE (D)	−0.71 ± 1.30 [−5.75, 1.50]		
UDVA (logMAR)	0.310 ± 0.176 [0.096, 0.824]		
CDVA (logMAR)	0.166 ± 0.073 [0.000, 0.301]		
UNVA (logMAR)	0.640 ± 0.375 [0.000, 1.301]		

	**IOLMaster 700**	**Argos**	**p** **value**

AL (mm)	25.38 ± 1.34 [22.76, 29.27]	25.36 ± 1.29 [22.78, 29.12]	0.031
ACD (mm)	3.34 ± 0.26 [2.83, 4.17]	3.44 ± 0.25 [2.95, 4.23]	< 0.001
Steep K (D)	40.78 ± 2.45 [34.97, 45.83]	41.46 ± 2.63 [34.91, 45.97]	< 0.001
Flat K (D)	40.08 ± 2.44 [33.41, 44.73]	40.64 ± 2.65 [32.99, 45.09]	< 0.001

*Note:* K, conventional keratometry.

Abbreviations: ACD, anterior chamber depth; AL, axial length; CDVA, corrected distance visual acuity; SE, Spherical equivalent; UDVA, uncorrected distance visual acuity; UNVA, uncorrected near visual acuity.

Figure 1 illustrates the distribution of UDVA and CDVA, SE, and refractive astigmatism at 6 months postoperatively. Toric IOLs were implanted in 58 of 94 eyes (61.7%) to correct preexisting corneal astigmatism. Postoperative visual outcomes and refraction outcomes are summarized in Table 2.

**FIGURE 1:**
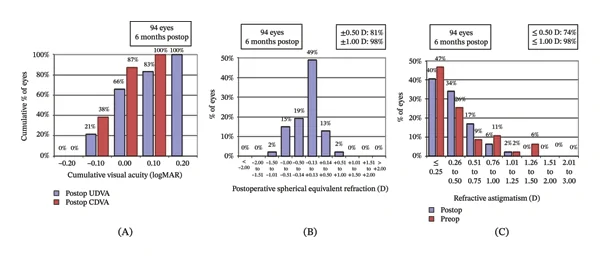
Postoperative cumulative monocular visual acuity and refractive outcomes at 6 months. (A) Cumulative visual acuity (logMAR), (B) spherical equivalent refraction, (C) refractive astigmatism.

**TABLE 2:** Monocular visual acuities (logMAR) and refractive outcomes at postoperative 1, 3, and 6 months in patients implanted with Clareon PanOptix.

	1 month	3 months	6 months
	Mean ± SD	Mean ± SD	Mean ± SD
UDVA (logMAR)	0.034 ± 0.093	0.031 ± 0.090	0.028 ± 0.087
CDVA (logMAR)	−0.014 ± 0.050	−0.022 ± 0.049	−0.023 ± 0.048
UIVA (logMAR)	0.161 ± 0.136	0.182 ± 0.133	0.177 ± 0.130
DCIVA (logMAR)	0.148 ± 0.094	0.144 ± 0.092	0.140 ± 0.091
UNVA (logMAR)	0.100 ± 0.142	0.095 ± 0.139	0.093 ± 0.137
DCNVA (logMAR)	0.082 ± 0.121	0.076 ± 0.119	0.074 ± 0.117
Sphere (D)	0.07 ± 0.41	0.07 ± 0.40	0.07 ± 0.40
Cylinder (D)	−0.45 ± 0.33	−0.42 ± 0.31	−0.42 ± 0.31
SE (D)	−0.15 ± 0.39	−0.14 ± 0.38	−0.14 ± 0.38

Abbreviations: CDVA, corrected distance visual acuity; DCIVA, distance‐corrected intermediate visual acuity; DCNVA, distance‐corrected near visual acuity; SE, Spherical equivalent; UDVA, uncorrected distance visual acuity; UIVA, uncorrected intermediate visual acuity; UNVA, uncorrected near visual acuity.

The lowest PE was observed when the IOLMaster 700 was used in conjunction with Barrett True‐K with TK, yielding a mean arithmetic PE of −0.071 ± 0.437 D, with a corresponding mean absolute error (MAE) of 0.338 ± 0.282 D and a median absolute error (MedAE) of 0.240 D [IQR, 0.130–0.443 D]. With the IOLMaster 700, the mean arithmetic PE was −0.114 ± 0.471 D for Barrett True‐K with CK (MAE: 0.388 ± 0.285 D; MedAE: 0.288 D [IQR, 0.169–0.576 D]); 0.262 ± 0.610 D for Haigis‐L with TK (MAE: 0.468 ± 0.468 D; MedAE: 0.375 D [IQR, 0.215–0.595 D]); and −0.302 ± 0.594 D for Haigis‐L with CK (MAE: 0.479 ± 0.459 D; MedAE: 0.385 D [IQR, 0.115–0.720 D]). With the Argos, the mean arithmetic PE was −0.318 ± 0.449 D for Barrett True‐K with TK (MAE: 0.464 ± 0.291 D; MedAE: 0.423 D [IQR, 0.228–0.583 D]); and −0.099 ± 0.507 D for Barrett True‐K with CK (MAE: 0.392 ± 0.332 D; MedAE: 0.265 D [IQR, 0.134–0.515 D]).

Figure 2 shows the prediction accuracy with the two biometers. With the IOLMaster 700, the Barrett True‐K with TK yielded the highest percentage of eyes within ±0.50 D (78.7%), followed by Haigis‐L with TK (73.3%), Barrett True‐K with CK (68.1%), and Haigis‐L with CK (65.2%). The mean arithmetic PE differed between TK and CK for Haigis‐L (mean difference = 0.564 ± 0.310 D; *p* < 0.001), but not for the Barrett True‐K formula (*p* = 0.267). With the Argos, Barrett True‐K with CK had a higher observed ±0.50 D rate than TK (71.7% vs 58.7%); the arithmetic PE also differed (mean difference = −0.227 ± 0.237 D; *p* < 0.001).

**FIGURE 2:**
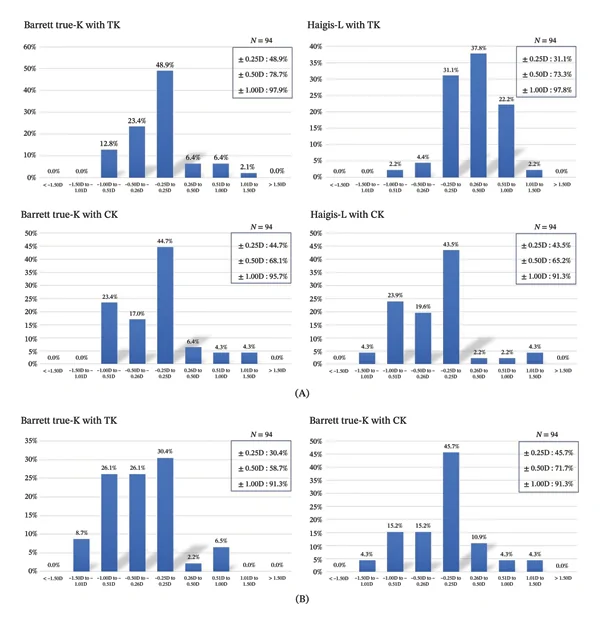
Distribution of refractive prediction errors at 6 months postoperatively. Bars indicate the percentage of eyes within each prediction error range, and the inset boxes show the cumulative percentage of eyes with prediction errors within ±0.25 D, ±0.50 D, and ±1.00 D. (A) Barrett true‐K and haigis‐L formulas with IOLMaster 700 measurements, using total keratometry (TK) or conventional keratometry (CK). (B) Barrett true‐K formula with argos measurements, using TK incorporating pentacam‐measured posterior corneal astigmatism or CK. Prediction error was defined as the actual postoperative spherical equivalent minus the predicted spherical equivalent. D, diopters.

When Barrett True‐K with TK was used, the mean arithmetic PE differed significantly between the IOLMaster 700 and Argos (mean difference = −0.260 ± 0.266 D; *p* < 0.001). In contrast, no significant difference was observed when both used CK (*p* = 0.818). When the combinations with the highest observed ±0.50 D rates on each device were compared—Barrett True‐K with TK on the IOLMaster 700 and Barrett True‐K with CK on the Argos—the mean arithmetic PE did not differ significantly (*p* = 0.400).

Table 3 displays the correlations between preoperative corneal HOAs and postoperative VA that remained significant after Holm–Bonferroni correction, stratified by analysis diameter. UDVA was associated with HOA RMS and NS‐HOA RMS at a 3.0‐mm diameter, whereas DCNVA was associated with HOA RMS and/or NS‐HOA RMS at the 4.0‐, 5.0‐, and 6.0‐mm diameters, and UNVA with both parameters at the 5.0‐mm diameter. The complete unadjusted correlation matrix across all analysis diameters is provided in Supporting Table 2.

**TABLE 3:** Spearman correlations (*ρ*) between preoperative corneal higher‐order aberrations and postoperative visual acuity remaining significant after Holm–Bonferroni correction, by analysis diameter.

mm	HOA parameter	Spearman’s rank correlation coefficient					
		UDVA	CDVA	UIVA	DCIVA	UNVA	DCNVA
3.0	HOA RMS	*ρ*: +0.373 *p* ^∗^ = 0.045					
	Primary Spherical Aberration						
	Non spherical HOA RMS	*ρ*: +0.373 *p* ^∗^ = 0.045					

4.0	HOA RMS						*ρ*: +0.367 *p* ^∗^ = 0.048
	Primary Spherical Aberration						
	Non spherical HOA RMS						*ρ*: +0.360 *p* ^∗^ = 0.048

5.0	HOA RMS					*ρ*: +0.354 *p* ^∗^ = 0.048	*ρ*: +0.385 *p* ^∗^ = 0.024
	Primary Spherical Aberration						
	Non spherical HOA RMS					*ρ*: +0.367 *p* ^∗^ = 0.048	*ρ*: +0.421 *p* ^∗^ = 0.015

6.0	HOA RMS						
	Primary Spherical Aberration						
	Non spherical HOA RMS						*ρ*: +0.376 *p* ^∗^ = 0.042

*Note:* Only correlations remaining statistically significant after Holm–Bonferroni correction are shown. *p*‐value was adjusted using the Holm–Bonferroni method within each analysis diameter.

Abbreviations: CDVA, corrected distance visual acuity; DCIVA, distance corrected intermediate visual acuity; DCNVA, distance corrected near visual acuity; UDVA, uncorrected distance visual acuity; UIVA, uncorrected intermediate visual acuity; UNVA, uncorrected near visual acuity.

Assessment of postoperative spectacle dependence revealed that no patients required spectacles for distance or intermediate vision (Supporting Table 3). Although 12 patients (25.5%) reported rarely needing spectacles for near vision when tested monocularly, none required spectacles for near visual tasks under binocular viewing conditions in daily life.

The postoperative questionnaire responses on subjective satisfaction are summarized in Supporting Table 4. No patients reported being dissatisfied or very dissatisfied. The mean satisfaction score was 3.09 ± 0.62 on a 0–4 scale. After adjustment for multiple comparisons, there was no meaningful correlation with various HOAs (adjusted *p* > 0.05).

Postoperative visual disturbances, including their frequency and severity, are presented in Supporting Table 5. Visual disturbance scores were not significantly correlated with postoperative UDVA or CDVA. Moderate or greater symptoms (grade ≥ 3) were reported in 5 patients (10.6%) for halo and 4 patients (8.5%) for starburst, whereas no patients reported moderate or greater glare. Importantly, none of the dysphotopsia symptoms required additional medical or surgical intervention, including refractive enhancement, IOL exchange, or IOL repositioning.

Table 4 summarizes the correlations between preoperative corneal HOAs and postoperative visual disturbances that remained statistically significant after Holm–Bonferroni correction, stratified by analysis diameter. Significant associations were concentrated at the 5.0‐ and 6.0‐mm analysis diameters, where HOA RMS and primary SA were positively correlated with blurred vision, hazy vision, and halos (*ρ* = +0.368 to +0.544), and NS‐HOA RMS was correlated with blurred vision. At the 4.0‐mm diameter, primary SA was correlated with blurred vision and glare.

**TABLE 4:** Spearman correlations (*ρ*) between preoperative corneal higher‐order aberrations and postoperative visual disturbances remaining significant after Holm–Bonferroni correction, by analysis diameter.

mm	HOA parameter	Blurred vision	Hazy vision	Halo	Glare	Starburst
3.0	HOA RMS					
	Primary Spherical Aberration					
	Non spherical HOA RMS					

4.0	HOA RMS					
	Primary Spherical Aberration	*ρ*: +0.494 *p* = 0.006			*ρ*: +0.365 *p* = 0.048	
	Non spherical HOA RMS					

5.0	HOA RMS	*ρ*: +0.532 *p* = <0.001	*ρ*: +0.394 *p* = 0.020	*ρ*: +0.368 *p* = 0.048	*ρ*: +0.400 *p* = 0.027	
	Primary Spherical Aberration	*ρ*: +0.536 *p* = <0.001	*ρ*: +0.420 *p* = 0.018	*ρ*: +0.379 *p* = 0.045		
	Non spherical HOA RMS	*ρ*: +0.409 *p* = 0.007				

6.0	HOA RMS	*ρ*: +0.434 *p* = 0.012	*ρ*: +0.436 *p* = 0.048	*ρ*: +0.464 *p* = 0.004		
	Primary Spherical Aberration	*ρ*: +0.420 *p* = 0.012	*ρ*: +0.481 *p* = 0.003	*ρ*: +0.544 *p* = <0.001		
	Non spherical HOA RMS	*ρ*: +0.371 *p* = 0.016				

*Note:* Only correlations remaining statistically significant after Holm–Bonferroni correction are shown. *p*‐value was adjusted using the Holm–Bonferroni method within each analysis diameter.

## 4. Discussion

To the best of our knowledge, this is the first study to evaluate the clinical efficacy and refractive predictability of CP trifocal IOL implantation in eyes with previous myopic LRS. Because these patients often expect to maintain spectacle independence after cataract surgery, multifocal IOLs have been considered a viable option [1, 3, 19]. However, altered corneal geometry and increased corneal HOAs after LRS can compromise both the accuracy of IOL power calculation and postoperative visual quality [18, 20, 21]. Accordingly, this study assessed four interrelated domains of clinical performance for the CP: visual and refractive outcomes; accuracy of IOL power calculations; associations between corneal HOAs and visual outcomes or patient‐reported visual quality; and patient‐reported outcomes.

The CP provided functional vision across far, intermediate, and near distances at 6 months postoperatively. Most patients achieved sufficient vision for daily activities without spectacles, with outcomes comparable to or better than those reported for other trifocal IOLs in post‐LRS eyes [3, 12, 19, 21]. Nonetheless, the proportion of eyes achieving excellent unaided distance acuity suggested a clinically meaningful “post‐LRS penalty.” In our study, 83% of eyes achieved a UDVA of 0.1 logMAR or better, which appears lower than that reported in a previous study of virgin eyes implanted with the same IOL [22]. Therefore, post‐LRS patients considering multifocal IOLs should be counseled that, although functional uncorrected vision is achievable, the highest level of VA may be less attainable than in virgin eyes.

Accurate prediction of refractive outcomes in post‐LRS eyes remains challenging [23]. At 6 months, 81% of eyes had a postoperative SE within ±0.50 D of emmetropia, exceeding previously reported rates for other trifocal IOLs in this population, including 73% with the AP, 65% with the FineVision, and 47.6% with the AT LISA tri 839MP [3, 12, 19]. Such differences may reflect the IOL power calculation method, inclusion of posterior corneal measurements, biometry accuracy for anterior corneal irregular astigmatism and K value variability, and surgeon‐related factors.

A notable finding was the influence of TK‐based IOL power calculation. With the IOLMaster 700, Barrett True‐K with TK increased ±0.50 D rates vs CK (78.7% vs 68.1%), and Haigis‐L with TK outperformed CK (73.3% vs 65.2%), consistent with previous reports [24]. Myopic LRS flattens the anterior corneal surface, thereby disrupting the normal physiological ratio between anterior and posterior curvature. CK‐based formulas assume a fixed ratio and estimate posterior corneal power, leading to a “keratometric index error” in post‐LRS eyes that may overestimate total corneal power and cause a hyperopic refractive surprise [25]. TK may therefore reduce keratometric index error by incorporating posterior corneal information more directly in post‐LRS eyes [24, 26].

Significant differences in K, ACD, and AL were observed between the IOLMaster 700 and Argos. Differences in AL may partly reflect the distinct optical models used for AL calculation: the Argos applies segmented refractive indices, whereas the IOLMaster 700 uses a single equivalent refractive index [27, 28]. In contrast, differences in K and ACD are more likely related to device‐specific measurement locations and algorithms [29, 30]. Notably, incorporation of posterior corneal astigmatism obtained from Pentacam into the Barrett True‐K calculation for Argos reduced predictive accuracy in our cohort. This does not contradict the general superiority of TK demonstrated with the IOLMaster 700; rather, it suggests that external incorporation of posterior corneal data without device‐specific validation may introduce calibration errors. These findings highlight that IOL power calculation should be regarded as an integrated system—biometer, measurement protocol, and calculation formula. A mix‐and‐match approach, using different devices without validated conversion factors, may introduce unanticipated errors [31–33]. In addition, refractive prediction accuracy depends on biometer‐specific formulas and on how altered corneal optical zones are measured [29, 30]. Accordingly, additional studies are needed to clarify how the relationship between keratometric measurement location and central corneal curvature, as well as the anterior and posterior corneal curvature ratio, influences refractive prediction in post‐LRS eyes.

Beyond biometry, corneal aberrations in post‐LRS eyes should be assessed at multiple analysis diameters. The effect of myopic correction on SA depends on the analyzed diameter. In our previous study, after myopic LRS using a 6.5‐mm optical zone, postoperative SA increased mainly when analyzed at larger diameters, with a correction‐dependent increase evident only at analysis diameters of 6 mm or larger, whereas SA analyzed at smaller diameters changed little or even decreased [34]. Therefore, the conventional 6‐mm SA metric does not necessarily represent the aberration within a smaller, pupil‐relevant diameter after LRS. This explains why even eyes with long AL could show SA at a 4.0‐mm analysis diameter within our predefined range and supports aberration assessment at multiple analysis diameters rather than at a single diameter in post‐LRS eyes.

Consistent with this concept, HOA analysis at multiple diameters in the present study provided additional insight into postoperative visual quality. Increased HOA RMS and NS‐HOA RMS at the 3.0‐mm analysis diameter were correlated with poorer postoperative UDVA, whereas elevated HOA RMS and NS‐HOA RMS at larger analysis diameters (4.0–6.0 mm) were associated with reduced near VA, and HOA RMS and primary SA, predominantly at the 5.0‐ and 6.0‐mm diameters, were associated with visual disturbances. These findings suggest that aberrations analyzed at smaller diameters (3.0‐mm), potentially relevant under photopic conditions, may influence high‐contrast VA (UDVA). In contrast, aberrations analyzed at larger diameters may become functionally relevant under scotopic conditions and contribute to photic phenomena. In line with our previous work [34, 35], these observations support a multidiameter approach for interpreting corneal HOAs in post‐LRS eyes. The clinical value of this approach lies in moving beyond a single global HOA parameter (e.g., total HOA RMS at the 6.0‐mm analysis diameter) and supporting more individualized patient counseling. For instance, eyes with elevated SA at larger analysis diameters may achieve good distance acuity yet experience photic phenomena under dim illumination that could affect subjective visual quality. Such a stratified approach may facilitate expectation management in post‐LRS patients receiving presbyopia‐correcting IOLs. Nevertheless, these associations should be considered hypothesis‐generating and require prospective validation.

Our results are consistent with several previous studies linking corneal HOAs to postoperative visual performance [18, 36, 37]. By contrast, other studies have identified UDVA as the primary determinant of satisfaction rather than HOAs [38]. This discrepancy may arise from unique features of post‐LRS eyes. The interaction between diffractive multifocal optics of the CP and surgically altered corneal HOA profiles may contribute to subtle image quality changes that can influence subjective satisfaction. Accordingly, while UDVA may be the dominant correlate of satisfaction in the general cataract population, corneal HOAs may play an important contributory role in visual quality in post‐LRS eyes.

Patient‐reported outcomes were favorable. Patients demonstrated high spectacle independence and a mean satisfaction score of 3.09 ± 0.62 on a 0–4 scale and no reported dissatisfaction. This favorable subjective outcome, despite the post‐LRS penalty observed in some objective parameters, may be partly attributable to protocol‐driven patient selection, using our routine aberration‐based screening criteria (described in the Methods). Indeed, the mean 4.0‐mm HOA RMS in included eyes (0.224 ± 0.099 μm) was well below the 0.6‐μm threshold. Dysphotopsia symptoms, particularly halos and glare, were generally mild and required no additional intervention. Blurred and hazy vision were more frequent, suggesting that visual quality complaints in post‐LRS eyes may not be fully captured by dysphotopsia symptoms alone. These symptoms were not correlated with postoperative UDVA but were associated with corneal HOAs at larger analysis diameters, supporting the relevance of preoperative HOA assessment in post‐LRS eyes. Furthermore, the absence of intervention or dissatisfaction supports good tolerance of the CP optical design in this population.

This study has several limitations. First, although the patient‐reported outcomes were collected prospectively, clinical and biometric data were analyzed retrospectively at a single center. Second, the relatively small sample size and the absence of a direct comparison group—either eyes without prior LRS or eyes implanted with another multifocal IOL—limit the generalizability of the findings. In addition, because our findings derive from eyes selected through this aberration‐based protocol, they should not be generalized to post‐LRS eyes with aberration profiles outside these criteria. Third, pupillometry was not performed. Because the pupil serves as the effective aperture stop of the ocular optical system, corneal HOAs computed over fixed analysis diameters of 3.0 to 6.0 mm do not necessarily correspond to the wavefront error that actually reaches the retina under photopic, mesopic, or scotopic conditions. In particular, the correlations observed at the 5.0‐ and 6.0‐mm analysis diameters may have limited physiological relevance in eyes whose pupils do not dilate to these diameters. This possibility cannot be excluded given the mean age of our cohort (53.70 ± 5.28 years), as a mean pupil diameter of approximately 4.97 mm under low luminance (2.5 cd/m^2^) has been reported in individuals aged 52 years or older [39]. Nevertheless, myopic refractive status has been associated with relatively larger pupils under low‐luminance or mesopic conditions [39, 40], suggesting that larger analysis diameters may still be physiologically relevant in some eyes. The present multidiameter HOA findings should therefore be interpreted as exploratory associations, and future studies incorporating dynamic pupillometry are warranted. Fourth, detailed historical LRS data, including the ablation profile, optical zone diameter, centration, and pre‐LRS refraction, were largely unavailable because most procedures had been performed at outside institutions many years earlier. Consequently, ablation type had to be inferred from current corneal tomographic patterns, and outcomes could not be stratified by the magnitude of previous myopic correction [41]. Unmeasured variability in these original parameters may therefore have contributed to residual outcome variability.

Cobo‐Soriano et al. reported that eyes with greater previous myopic correction showed reduced refractive predictability and greater residual myopic error after trifocal IOL implantation, although postoperative VA remained favorable [41]. In that large consecutive series, however, no quantitative corneal aberration thresholds comparable to those used in our study were reported. Corneal irregularity is a major source of error in estimating true corneal power and predicting refractive outcomes in post‐LRS eyes [42]. The induction of corneal SA and coma increases in proportion to the magnitude of myopic correction [43], and when the optical zone is small, keratometric measurements may sample the transition zone outside the effective optical zone, further increasing the risk of a refractive surprise [25]. Accordingly, eyes with lower irregular astigmatism would be expected to achieve better refractive predictability, and favorable outcomes have been reported when corneal aberration‐based screening criteria comparable to ours were applied [1]. The favorable refractive outcomes in our cohort, despite the unavailability of historical LRS data, may therefore be at least partly attributable to our aberration‐based patient selection.

Future large‐scale, prospective, multicenter studies are needed to validate the relative performance of the CP and the clinical applicability of the multidiameter HOA approach. Stratification by predefined aberration profiles at specific analysis diameters may help determine whether this approach can evolve into an HOA‐based algorithm for customized IOL selection in post‐LRS patients.

Our findings indicate that the CP trifocal IOL is an effective option for presbyopia in post‐myopic LRS eyes meeting predefined preoperative corneal aberration criteria. However, in eyes with surgically altered corneas, the IOL itself is only one component of a successful outcome. Refractive predictability and postoperative visual outcomes are likely to depend on a combined clinical strategy that incorporates modern biometry with the formula–keratometry combination best suited to each device—Barrett True‐K with TK for the IOLMaster 700 or Barrett True‐K with CK for the Argos, which showed comparable predictive accuracy in this study—together with assessment of corneal aberrations at multiple analysis diameters to refine patient selection and set realistic expectations.

**Nomenclature**
AL: Axial length
ACD: Anterior chamber depth
AP: AcrySof PanOptix
CDVA: Corrected distance visual acuity
CK: Conventional keratometry
CP: Clareon PanOptix
D: Diopter
DCIVA: Distance‐corrected intermediate visual acuity
DCNVA: Distance‐corrected near visual acuity
HOA: Higher‐order aberration
IOL: Intraocular lens
IQR: Interquartile range
K: Keratometry
LASIK: Laser‐assisted in situ keratomileusis
LogMAR: Logarithm of the minimum angle of resolution
LRS: Laser refractive surgery
LT: Lens thickness
MedAE: Median absolute error
MAE: Mean absolute error
NS‐HOA RMS: Nonspherical higher‐order aberration root mean square
OCT: Optical coherence tomography
PE: Prediction error
PRK: Photorefractive keratectomy
RMS: Root mean square
SA: Spherical aberration
SE: Spherical equivalent
TK: Total keratometry
UDVA: Uncorrected distance visual acuity
UIVA: Uncorrected intermediate visual acuity
UNVA: Uncorrected near visual acuity
VA: Visual acuity

## Funding

This study was funded by Alcon Research LLC, Fort Worth, TX, USA, and Alcon Korea Ltd., Seoul, South Korea (Grant ID 89017305).

## Disclosure

The funding organization had no role in the design or conduct of this research.

## Ethics Statement

This study was approved by the Institutional Review Board of First Samsung Eye Clinic (IRB No. FSEC‐202311‐HR010‐02) and adhered to the tenets of the Declaration of Helsinki. Written informed consent was obtained from all participants for the prospective component of the study.

## Conflicts of Interest

The authors declare no conflicts of interest.

## Supporting Information

Additional supporting information can be found online in the Supporting Information section.

## Supporting information


**Supporting Information 1** Supporting Table 1. Preoperative corneal higher‐order aberrations by analysis diameter. Supporting Table 2. Unadjusted spearman correlation between preoperative corneal higher‐order aberrations and postoperative visual acuity by analysis diameter. UDVA, uncorrected distance visual acuity; CDVA, corrected distance visual acuity; UNVA, uncorrected near visual acuity; DCNVA, distance corrected near visual acuity, ^∗^ *p* < 0.05. Supporting Table 3. Questionnaire for spectacle dependence at 6 months postoperatively (*N* = 47 Patients). Supporting Table 4. Postoperative questionnaire for subjective satisfaction after cataract surgery (*N* = 47 patients). Supporting Table 5. Postoperative questionnaire for visual disturbances after cataract surgery (*N* = 47 patients).

**Supporting Information 2** STROBE checklist.

## Data Availability

The data supporting the findings of this study are not publicly available due to patient privacy and ethical restrictions. De‐identified data may be made available from the corresponding author upon reasonable request and with appropriate institutional approval, where applicable.
