# Supplementary material for: Hydrophobic Acrylic Trifocal IOL in Post–Laser Refractive Surgery Eyes: Visual Performance, Predictability, and Patient‐Reported Outcomes
Source: J Ophthalmol. 2026 Oct 21;2026:7762608. doi: 10.1155/joph/7762608 (PMC13685514; doi:10.1155/joph/7762608)
Supplement: Supplementary file 1 [file JOPH-2026-7762608-s002.docx]

Supplementary Table 1. Preoperative Corneal Higher-Order Aberrations by Analysis Diameter

| **Aberration** | **3.0 mm** | **4.0 mm** | **5.0 mm** | **6.0 mm** |
| --- | --- | --- | --- | --- |
|  |  |  |  |  |
| **Vertical Coma** | -0.016 ± 0.077 | -0.027 ± 0.128 | -0.015 ± 0.217 | 0.043 ± 0.389 |
| **Horizontal Coma** | 0.014 ± 0.046 | 0.040 ± 0.088 | 0.086 ± 0.171 | 0.140 ± 0.309 |
| **Coma RMS** | 0.077 ± 0.050 | 0.131 ± 0.095 | 0.222 ± 0.183 | 0.396 ± 0.330 |
| **Vertical Trefoil** | 0.006 ± 0.053 | -0.002 ± 0.092 | -0.032 ± 0.136 | -0.083 ± 0.189 |
| **Oblique Trefoil** | -0.007 ± 0.036 | -0.015 ± 0.051 | -0.023 ± 0.075 | -0.026 ± 0.126 |
| **Trefoil RMS** | 0.054 ± 0.034 | 0.087 ± 0.060 | 0.139 ± 0.078 | 0.218 ± 0.104 |
| **Primary Spherical Aberration** | 0.003 ± 0.037 | 0.037 ± 0.070 | 0.170 ± 0.093 | 0.519 ± 0.191 |
| **Absolute Primary Spherical**  **Aberration** | 0.024 ± 0.027 | 0.064 ± 0.046 | 0.170 ± 0.093 | 0.519 ± 0.191 |
| **HOA RMS** | 0.118 ± 0.053 | 0.224 ± 0.099 | 0.410 ± 0.172 | 0.811 ± 0.324 |
| **Non spherical HOA RMS** | 0.114 ± 0.050 | 0.212 ± 0.095 | 0.364 ± 0.167 | 0.607 ± 0.298 |

Supplementary Table 2. Unadjusted Spearman Correlations Between Preoperative Corneal Higher-Order Aberrations and Postoperative Visual Acuity by Analysis Diameter

| mm | Aberration | UDVA | | CDVA | | UNVA | | DCNVA | |
| --- | --- | --- | --- | --- | --- | --- | --- | --- | --- |
|  |  | Spearman ρ | p-value | Spearman ρ | p-value | Spearman ρ | p-value | Spearman ρ | p-value |
| 3.0 mm | Vertical Trefoil | +0.035 | 0.827 | +0.050 | 0.756 | +0.126 | 0.426 | +0.066 | 0.679 |
|  | Vertical Coma | –0.104 | 0.513 | +0.102 | 0.521 | +0.084 | 0.595 | -0.004 | 0.980 |
|  | Horizontal Coma | –0.111 | 0.482 | –0.075 | 0.637 | -0.061 | 0.699 | -0.043 | 0.787 |
|  | Oblique Trefoil | +0.149 | 0.345 | +0.332 | 0.032* | -0.001 | 0.995 | +0.091 | 0.566 |
|  | Trefoil RMS | +0.331 | 0.032* | +0.040 | 0.801 | +0.297 | 0.056 | +0.255 | 0.103 |
|  | Coma RMS | +0.294 | 0.059 | +0.129 | 0.415 | +0.103 | 0.517 | +0.151 | 0.341 |
|  | HOA RMS | +0.373 | 0.015* | +0.182 | 0.249 | +0.279 | 0.074 | +0.239 | 0.128 |
|  | Primary Spherical Aberration | +0.023 | 0.883 | –0.269 | 0.084 | +0.102 | 0.522 | -0.011 | 0.947 |
|  | Non-Spherical HOA RMS | +0.373 | 0.015* | +0.168 | 0.289 | +0.246 | 0.116 | +0.214 | 0.174 |
| 4.0 mm | Vertical Trefoil | –0.046 | 0.770 | –0.102 | 0.521 | +0.034 | 0.832 | -0.052 | 0.744 |
|  | Vertical Coma | –0.144 | 0.364 | +0.115 | 0.468 | +0.082 | 0.607 | -0.007 | 0.967 |
|  | Horizontal Coma | –0.053 | 0.741 | -0.080 | 0.616 | -0.074 | 0.643 | -0.033 | 0.837 |
|  | Oblique Trefoil | +0.124 | 0.435 | +0.292 | 0.060 | -0.029 | 0.855 | +0.093 | 0.557 |
|  | Trefoil RMS | +0.309 | 0.026* | +0.047 | 0.769 | +0.274 | 0.079 | +0.263 | 0.093 |
|  | Coma RMS | +0.237 | 0.130 | +0.274 | 0.079 | +0.162 | 0.307 | +0.272 | 0.082 |
|  | HOA RMS | +0.344 | 0.026* | +0.182 | 0.248 | +0.351 | 0.023* | +0.367 | 0.016* |
|  | Primary Spherical Aberration | +0.059 | 0.711 | -0.191 | 0.226 | +0.182 | 0.249 | +0.030 | 0.852 |
|  | Non-Spherical HOA RMS | +0.369 | 0.016* | +0.210 | 0.182 | +0.339 | 0.028* | +0.360 | 0.019* |
| 5.0 mm | Vertical Trefoil | –0.165 | 0.295 | –0.284 | 0.068 | -0.165 | 0.329 | -0.271 | 0.083 |
|  | Vertical Coma | –0.322 | 0.038* | –0.053 | 0.739 | -0.007 | 0.964 | -0.072 | 0.647 |
|  | Horizontal Coma | +0.029 | 0.854 | –0.099 | 0.535 | -0.068 | 0.671 | -0.005 | 0.977 |
|  | Oblique Trefoil | +0.143 | 0.365 | +0.256 | 0.102 | -0.026 | 0.870 | +0.050 | 0.755 |
|  | Trefoil RMS | +0.369 | 0.016* | +0.238 | 0.129 | +0.254 | 0.104 | +0.337 | 0.029* |
|  | Coma RMS | +0.093 | 0.558 | +0.067 | 0.673 | +0.299 | 0.054 | +0.436 | 0.004* |
|  | HOA RMS | +0.182 | 0.248 | +0.096 | 0.547 | +0.354 | 0.021* | +0.385 | 0.012* |
|  | Primary Spherical Aberration | +0.089 | 0.576 | –0.061 | 0.702 | +0.170 | 0.282 | +0.074 | 0.642 |
|  | Non-Spherical HOA RMS | 0.237 | 0.131 | +0.149 | 0.346 | +0.367 | 0.017* | +0.321 | 0.005* |
| 6.0 mm | Vertical Trefoil | –0.081 | 0.611 | –0.288 | 0.065 | -0.233 | 0.137 | -0.318 | 0.040* |
|  | Vertical Coma | –0.249 | 0.112 | +0.022 | 0.890 | +0.063 | 0.693 | +0.038 | 0.809 |
|  | Horizontal Coma | +0.091 | 0.557 | -0.065 | 0.682 | -0.038 | 0.810 | +0.082 | 0.607 |
|  | Oblique Trefoil | +0.110 | 0.489 | +0.116 | 0.465 | +0.057 | 0.817 | -0.004 | 0.979 |
|  | Trefoil RMS | +0.238 | 0.129 | +0.283 | 0.069 | +0.100 | 0.527 | +0.331 | 0.033* |
|  | Coma RMS | –0.036 | 0.821 | -0.002 | 0.992 | +0.317 | 0.041* | +0.390 | 0.011* |
|  | HOA RMS | +0.018 | 0.912 | +0.086 | 0.590 | +0.294 | 0.059 | +0.311 | 0.045* |
|  | Primary Spherical Aberration | +0.061 | 0.701 | +0.181 | 0.250 | +0.206 | 0.191 | +0.168 | 0.288 |
|  | Non-Spherical HOA RMS | 0.063 | 0.694 | -0.030 | 0.850 | +0.328 | 0.034* | +0.376 | 0.014* |

UDVA, uncorrected distance visual acuity; CDVA, corrected distance visual acuity; UNVA, uncorrected near visual acuity; DCNVA, distance corrected near visual acuity, * p < 0.05

Supplementary Table 3. Questionnaire for spectacle dependence at 6 months postoperatively (N = 47 Patients)

| Score | Frequency | Distance | Intermediate | Near |
| --- | --- | --- | --- | --- |
|  |  | **N (%)** | **N (%)** | **N (%)** |
| 0 | Never | 47 (100.0) | 47 (100.0) | 35 (74.5) |
| 1 | Rarely | 0 (0.0) | 0 (0.0) | 8 (17.0) |
| 2 | Occasionally | 0 (0.0) | 0 (0.0) | 4 (8.5) |
| 3 | Frequently | 0 (0.0) | 0 (0.0) | 0 (0.0) |
| 4 | Always | 0 (0.0) | 0 (0.0) | 0 (0.0) |

Supplementary Table 4. Postoperative questionnaire for subjective satisfaction after cataract surgery (N = 47 Patients)

| Satisfaction Score | Description | Number of Respondents (n) | Percentage (%) |
| --- | --- | --- | --- |
| 0 | Very dissatisfied | 0 | 0.0 |
| 1 | Dissatisfied | 0 | 0.0 |
| 2 | Neutral | 7 | 14.9 |
| 3 | Satisfied | 29 | 61.7 |
| 4 | Very satisfied | 11 | 23.4 |
| Total | — | **47** | **100.0** |

Supplementary Table 5. Postoperative questionnaire for visual disturbances after cataract surgery (N = 47 Patients)

| **Symptom** | **Patients in each score (n)** | | | | | **Patients with  symptoms (%)** | **Score**  **(Mean ± SD)** |
| --- | --- | --- | --- | --- | --- | --- | --- |
|  | **0** | **1** | **2** | **3** | **4** |  |  |
| **Halo** | 21 | 17 | 4 | 5 | 0 | 55.3 | 0.85 ± 0.98 |
| **Glare** | 41 | 4 | 2 | 0 | 0 | 12.8 | 0.17 ± 0.48 |
| **Starburst** | 20 | 10 | 13 | 4 | 0 | 57.4 | 1.02 ± 1.03 |
| **Hazy Vision** | 18 | 6 | 9 | 13 | 1 | 61.7 | 1.43 ± 1.31 |
| **Blurred Vision** | 14 | 9 | 16 | 7 | 1 | 70.2 | 1.40 ± 1.14 |
| **Diplopia** | 0 | 0 | 0 | 0 | 0 | 0 | 0 |

Scores represent the mean severity rated on a 5-point scale (0 = none, 4 = always)
